# Our surgical procedure in acut thrombosis of stent graft using in treatment of pseudoaneurysm developing in popliteal anastomosis region of previous cross-over composite sequential femoro-popliteal bypass

**DOI:** 10.1186/1749-8090-8-S1-P116

**Published:** 2013-09-11

**Authors:** K Ergunes, E Celik, O Gokalp, H Iner, H Cakır, A Gurbuz

**Affiliations:** 1 Izmir Katip Celebi University Ataturk Training and Research Hospital, Department of Cardiovascular Surgery, Izmir, Turkey

## Background

Occlusion of femoro-popliteal bypass graft represents a limb and life-threatening condition requiring emergency intervention.

## Methods

A 66-year-old man was admitted to our hospital on November 11, 2012. He had a painful, pulseless, and cold below-knee in right lower extremity. He had previous right cross-over composite sequential femoro-popliteal bypass. Angiography showed acute occlusion of cross-over composite femoro-popliteal bypass graft and stent graft used in treatment of pseudoaneurysm developing in popliteal anastomosis region of this graft. Proximal EPTFE graft was occluded with thrombus in arteriography.

## Results

Thrombectomy was performed with the 5F Fogarty balloon catheter and excessive thrombus was extracted. A thrombo-embolectomy was performed to the distal of popliteal artery, but thrombus was not detected in this region. The patient was performed a new interposition saphenous vein graft between the proximal EPTFE graft and distal popliteal artery. The patient received low-molecular weight heparin in postoperative period. He received warfarin and acetylsalicylic acid in follow-up. Right lower extremity pulses were palpable in postoperative period. Right lower extremity had good function in follow-up at 6 months.

## Conclusions

We showed importance of saphenous vein graft bypass and thrombectomy in a case of having acute occlusion of cross-over composite sequential femoro-popliteal bypass.

